# A Retrospective Review of Upper Gastrointestinal Bleed Outcomes During Hospital Admission While on Oral Anticoagulation

**DOI:** 10.7759/cureus.15061

**Published:** 2021-05-16

**Authors:** Nicolina Scibelli, Andrew Mangano, Kathleen Raynor, Sarah Wilson, Pratishtha Singh

**Affiliations:** 1 Internal Medicine, Grand Strand Medical Center, Myrtle Beach, USA; 2 Gastroenterology, Grand Strand Medical Center, Myrtle Beach, USA; 3 Statistics, HCA Healthcare, Brentwood, USA

**Keywords:** upper gastrointestinal bleed, ugib, direct-acting oral anticoagulants, apixaban, rivaroxaban, dabigatran, warfarin, ppi, proton-pump inhibitor

## Abstract

Introduction

Direct-acting oral anticoagulants (DOACs) are approved for stroke prevention in non-valvular atrial fibrillation and treatment of venous thromboembolism. Most recent guidelines recommend DOACs over warfarin for most diagnoses given their predictable pharmacodynamics, lack of required monitoring, and safety profile. Specific outcomes such as shock, acute renal failure, and blood transfusion requirement while on oral anticoagulation compared to no anticoagulation remain unknown in patients with upper gastrointestinal (GI) bleeds.

Methods

This retrospective study used the HCA Healthcare Enterprise Data Warehouse (EDW) to analyze 13,440 patients aged >18 years that were admitted with an upper GI bleed from January 2017 to December 2019. The patients were categorized based on oral anticoagulant (i.e. rivaroxaban, apixaban, dabigatran and warfarin). The control group was patients admitted with an upper GI bleed not on oral anticoagulation. We evaluated the severity of upper GI bleeds while on oral anticoagulation based on the outcomes: mortality rate, length of stay, acute renal failure, shock, and need for packed red blood cell transfusions (pRBC). Comorbid conditions assessed were coronary artery disease (CAD), chronic obstructive pulmonary disease (COPD), heart failure (HF), atrial fibrillation (AF), venous thromboembolism (VTE), peripheral vascular disease (PVD), tobacco abuse, alcohol abuse, and chronic kidney disease (CKD). Home use of proton pump inhibitors (PPI), aspirin, and P2Y12 inhibitors were also evaluated.

Results

Patients on a DOAC without home PPI have a mortality odds ratio of 3.066 with a confidence interval (CI) greater than 95% (1.48-6.26, p<0.05) compared to patients on a DOAC and home PPI. Patients on warfarin and no home PPI have a mortality odds ratio of 5.55 (95% CI (1.02-30.35), p<0.05) compared to those on warfarin with home PPI use. In the no anticoagulation group, those not on PPI have an odds ratio of 3.28 (95% CI (2.54-4.24), p<0.05) of death compared to home PPI use. There was no statistical difference in mortality between each DOAC and warfarin.

There was no difference in the presence of acute renal failure or shock when comparing each DOAC, warfarin, and no medication. For patients presenting with GI bleed, 0.8414 units of pRBC were transfused. Patients not on oral anticoagulation were found to have statistically significant decrease in pRBC transfusion if they did not report alcohol use, CKD, HF, AF, VTE, PVD. Patients on DOACs and alcohol use have an average pRBC transfusion count that is 0.922 units more than those without reported alcohol use (p=0.006). In the warfarin group, there was no statistical significance noted when comparing pRBC transfusions and also when comparing to baseline comorbidities.

Conclusion

The retrospective study leads us to conclude that overall, patients taking the DOACs or warfarin had no statistically significant increase in RBC transfusions, length of stay, shock, acute renal failure, or mortality rate compared to patients who were not on oral anticoagulation. Home PPI use was shown to lower odds of mortality in patients on anticoagulation who presented with upper GI bleeding. PPI use had no effect on the need for transfusion or length of stay in patients on anticoagulation. These results can help predict which patients are likely to have higher mortality based on the use of home PPIs.

## Introduction

Direct-acting oral anticoagulants (DOACs) are approved for stroke prevention in non-valvular atrial fibrillation and the treatment of venous thromboembolism [1-9]. DOACs have become the preferred oral anticoagulant over warfarin given their predictable pharmacodynamics, lack of required monitoring, and safety profile [2-9]. Gastrointestinal bleeding is a significant cause of morbidity and mortality among patients who have been initiated on oral anticoagulation [10]. Apixaban has been noted to have the most favorable GI safety profile when compared to both dabigatran and rivaroxaban [9]. Rivaroxaban has been associated with increased rate of GI bleed compared to other DOACs and warfarin but specific outcomes in GI bleeds such as length of stay and need for red blood cell transfusions (pRBC) transfusion have not yet been defined [2]. A meta-analysis revealed that without taking dose of individual DOACs into account, the rate of major gastrointestinal (GI) bleeding was similar for DOACs and warfarin [3]. A study performed at the Medical University of South Carolina from 2010 to 2016 showed that 61 outpatients taking DOACs with acute GI bleed had lower rates of hospitalizations and blood transfusions compared to warfarin [10]. Our study aimed to assess the outcomes in hospitalized patients on oral anticoagulants based on the endpoints of shock, acute renal failure, need for red blood cell transfusion, and length of stay. We also investigated home use of PPI use in this population. We endeavored to risk-stratify patients for admission to floor versus critical care units and the need for urgent endoscopy intervention and the determined effect of PPI use in this population.

## Materials and methods

Study Design

This retrospective study evaluated patient information from 15 hospitals in the Southeast region of the United States. Data were obtained from the HCA Healthcare EDW which included inpatient, laboratory and pharmacy claims coded with International Classification of Diseases (ICD) revision 10 during an inpatient hospital stay. This study was conducted in compliance with the HCA requirements and received an institutional review board (IRB) exempt determination through Centralized Algorithms for Research Rules on IRB exemptions (CARRIE). 

Cohort

Our cohort was created using patients 18 years and over who were admitted to the hospital with a diagnosis of an upper-GI bleed. Exclusion criteria were patients under 18 years old and patients who were currently on dialysis. The study included a total of 13,440. The study index date was defined as the date of hospitalization. Our dates ranged from January 1, 2017 to December 31, 2019 and patients were followed from index date to hospital discharge.

Outcomes and exposure coding

The outcomes were incidence of mortality, length of stay in the hospital, incidence of shock, need for red blood cell transfusion, and incidence of acute renal failure.

Covariates

At baseline (date of admission), for each patient, demographic, comorbid, clinical and pharmacy data were extracted. Demographic characteristics included age, sex, and race. Other variables examined included home proton pump inhibitor (PPI) use, home aspirin use, home P2Y12 inhibitor use, tobacco use, and alcohol abuse. For baseline comorbid conditions, we included coronary artery disease (CAD), chronic obstructive pulmonary disease (COPD), heart failure, atrial fibrillation (AF), hypertension (HTN), diabetes mellitus (DM), peripheral vascular disease (PVD), tobacco abuse, alcohol abuse, and chronic kidney disease (CKD). 

Statistical analysis

A logistic regression model was used to assess the probability of an event occurring given a list of predictor variables using coefficient estimates and odds ratios. Variables of mortality, development of shock, acute renal failure were assessed while controlling for age, sex, home PPI use, home aspirin use, home P2Y12 use, and comorbidities of CAD, COPD, heart failure, AF, HTN, DM, PVD, tobacco abuse, alcohol abuse, and CKD. Using this method, we compared the likelihood of these events occurring in patients on each different DOAC (apixaban, rivaroxaban, and dabigatran), warfarin, and no medication. This same procedure was enacted again when observing only the variable of mortality and using the same controls, but in this analysis, the comparison was between all DOACs, warfarin, and no medication. The grouping was further separated into those on a home PPI versus those who were not. In another analysis, linear regression models were used to analyze statistical significance (α=0.05) between length of stay and RBC transfusion requirements.

## Results

Of the 13,440 patients identified, 491 were on apixaban, 188 on rivaroxaban, 35 on dabigatran, 225 on warfarin, and 12,501 patients were not taking oral anticoagulation. Table 1 shows the baseline characteristics for each group. 

**Table 1:** Study demographics. Demographic and clinical characteristics, including age, sex, race, and ethnicity, as well as preexisting comorbid conditions, of all patients admitted for gastrointestinal bleeding. AC: anticoagulation; SD: standard deviation: PPI: proton pump inhibitor: HTN: hypertension: CAD: coronary artery disease; COPD: chronic obstructive pulmonary disease; VTE: venous thromboembolism; DVT: deep venous thrombosis; PE: pulmonary embolus; PVD: peripheral vascular disease; CKD: chronic kidney disease.

	Apixaban (N=491)	Rivaroxaban (N=188)	Dabigatran (N=35)	Warfarin (N=225)	No oral AC (N=12501)	Statistical analysis	p-value
Average age (years) (mean ± SD)	70.96±13.22	69.40±13.74	74.49±8.42	68.38±13.59	58.50±18.07	ANOVA	<0.0001
Sex: male	263 (53.56%)	103 (54.79%)	17 (48.57%)	140 (62.22%)	5980 (47.84%)	Chi-square	<0.0001
Sex: female	228 (46.44%)	85 (45.21%)	18 (51.43%)	85 (37.78%)	6521 (52.16%)		
Race (N): Caucasian	384 (78.21%)	152 (80.85%)	30 (85.71%)	184 (81.78%)	8523 (68.18%)	Fisher's Exact	<0.0001
Race (N): African American	98 (19.96%)	27 (14.36%)	3 (8.57%)	33 (14.67%)	3217 (25.73%)		
Race (N): other	9 (1.83%)	9 (4.79%)	2 (5.71%)	8 (3.56%)	761 (6.09%)		
Confounding variables							
Home aspirin use	171 (34.83%)	54 (28.72%)	14 (40%)	77 (34.22%)	1591 (12.73%)	Chi square	<0.0001
Home P2Y12 inhibitor	71 (14.46%)	19 (10.11%)	4 (11.43%)	18 (8%)	595 (4.76%)	Fisher's Exact	<0.0001
Home PPI use	307 (62.53%)	115 (61.17%)	21 (60%)	146 (64.89%)	4859 (38.87%)	Chi square	<0.0001
Past medical history (N)							
HTN	187 (38.09%)	86 (45.74%)	11 (31.43%)	87 (38.67%)	4701 (37.6%)	Chi square	<0.0001
CAD	175 (35.64%)	58 (30.85%)	16 (45.71%)	74 (32.89%)	1612 (12.89%)	Chi square	<0.0001
COPD	3 (.61%)	2 (1.06%)	0 (0%)	1 (.44%)	111 (0.89%)	Fisher's Exact	0.9685
Heart failure	102 (20.77%)	39 (20.74%)	7 (20%)	53 (23.56%)	744 (5.95%)	Fisher's Exact	<0.0001
Atrial fibrillation	294 (59.88%)	107 (56.91%)	25 (71.43%)	115 (51.11%)	786 (6.29%)	Fisher's Exact	<0.0001
History of VTE (chronic DVT + chronic PE)	72 (14.66%)	19 (10.11%)	0 (0%)	31 (13.78%)	149 (1.19%)	Fisher's Exact	<0.0001
PVD	25 (5.09%)	12 (6.38%)	0 (0%)	13 (5.78%)	221 (1.77%)	Fisher's Exact	<0.0001
CKD	130 (26.48%)	40 (21.28%)	14 (40%)	54 (24%)	1083 (8.66%)	Fisher's Exact	<0.0001
Diabetes mellitus	127 (25.97%)	40 (21.28%)	7 (20%)	52 (23.11%)	1083(8.66%)	Fisher’s Exact	<0.0001
Smoking: former	190 (38.7%)	63 (33.51%)	18 (51.43%)	75 (33.33%)	2431 (19.45%)	Chi Square	<0.0001
Smoking: current	65 (13.24%)	26 (13.83%)	5 (14.29%)	37 (16.44%)	2798 (22.38%)	Chi Square	<0.0001
Alcohol abuse	25 (5.09%)	11 (5.85%)	3 (8.57%)	17 (7.56%)	924 (7.39%)	Fisher's Exact	0.4598

Mortality

**Figure 1:**
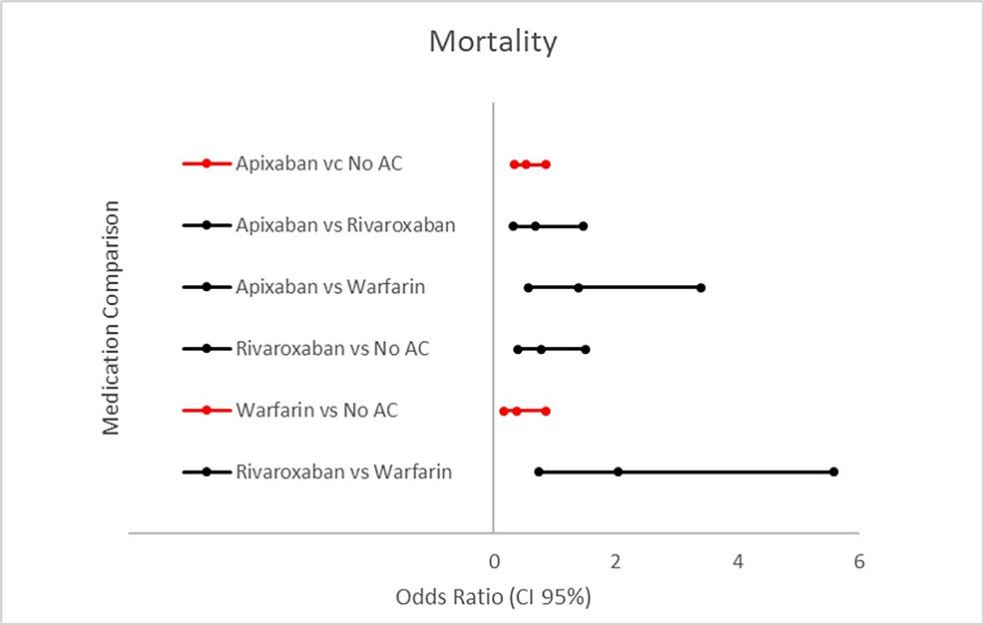
Odds ratio of mortality when comparing different DOACs, warfarin, and no AC. Forest plot comparing the mortality of our different DOACs, warfarin and, no AC. The comparisons are shown as an odds ratio. The middle marker represents the odds ratio, the lower value marker representing the lower limit of the 95% CI (confidence interval), and the higher value marker representing the upper limit of the 95% CI. The comparisons in red were found to be statistically significant. AC: anticoagulation.

Mortality for each group is listed in Table 2 and Figure 1. A total of 437 (3.25%) patients in our study population died during their inpatient stay. Patients on home apixaban were 1.89 times less likely to die when compared to patients on no home anticoagulation (odds ratio 0.529, 95% CI (0.329,0.852), p<0.05). Patients on home warfarin were 2.63 times less likely to die compared to those on no home anticoagulation (odds ratio 0.380, 95% CI (0.170,0.850) p<0.05). When comparing DOACs to one another and to warfarin, no statistical significance was detected. 

**Table 2:** Mortality, acute renal failure and shock in patients on DOACs vs. warfarin vs. no medications. Number of patients in each group and percentage mortality, acute renal failure and shock associated with each group. DOACs: direct-acting oral anticoagulants.

Category	Mortality, N (%)	Acute renal failure, N (%)	Shock, N (%)
DOACs	34 (4.76%)	226 (31.65%)	31 (4.3%)
Apixaban	23 (4.68%)	164 (33.40%)	24 (4.89%)
Rivaroxaban	11 (5.85%)	50 (26.60%)	5 (2.66%)
Dabigatran	0 (0%)	12 (34.29%)	2 (5.71%)
Warfarin	7 (3.11%)	66 (29.33%)	7 (3.1%)
No oral anticoagulation	396 (3.17%)	1777 (14.21%)	256 (2.0%)

**Figure 2:**
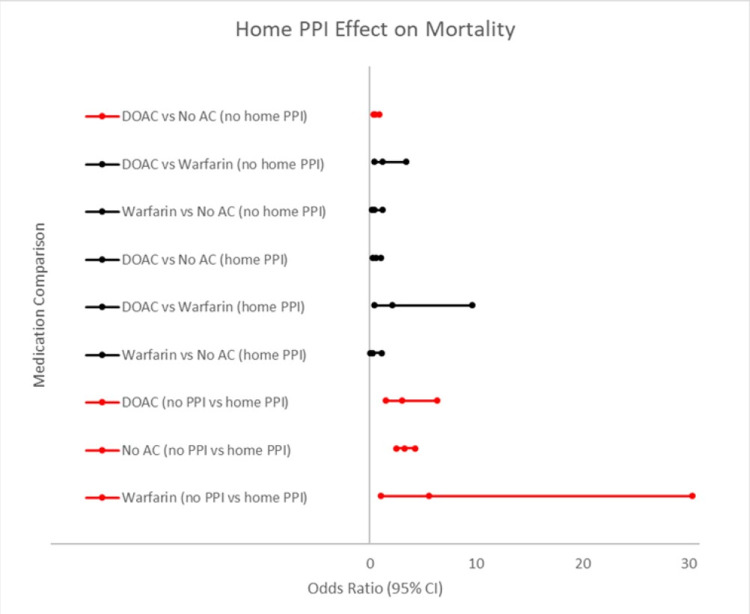
Odds ratio of mortality when comparing different DOACs, warfarin, and no AC with and without home PPI use. Forest plot comparing the effect of PPI on mortality between our different DOACs, warfarin and No AC. The comparison was shown as an odds ratio. The middle marker represents the odds ratio, the lower value marker representing the lower limit of the 95% CI, and the higher value marker representing the upper limit of the 95% CI. The comparisons in red were found to be statistically significant. DOAC: direct-acting oral anticoagulant; AC: anticoagulation; PPI: proton pump inhibitor.

Subanalysis revealed that home PPI use conveyed a mortality benefit for those both on oral anticoagulation and those not on oral anticoagulation (Figure 2). Patients on DOACs and not on home PPI were 1.83 times less likely to die than those not on PPI and no anticoagulation (95% CI (0.328-0.905), p<0.05). Patients with DOAC use and no home PPI had an odds ratio of death of 3.066 (95% CI (1.48-6.26), p<0.05) compared to patients on a DOAC and home PPI. Patients on warfarin and no home PPI had a mortality odds ratio of 5.55 (95% CI (1.02-30.35), p<0.05) compared to those on warfarin and home PPI. In the no anticoagulation group, those not on PPI had an odds ratio of 3.28 (95% CI (2.54-4.24), p<0.05) of death compared to home PPI use. Overall, patients on home PPI had an overall decreased mortality when compared to patients not on PPI. Home aspirin or P2Y12 inhibitor use had no statistical significance in regards to mortality when comparing each anticoagulant. 

Renal failure

Out of 13,440 patients in our study population, a total of 2,069 (15.39%) patients presented with acute renal failure and upper-GI bleed. Acute renal failure was either present on admission or during hospital admission. Table 2 shows the incidence of acute renal failure by the anticoagulation group. There was no statistical significance when comparing each anticoagulation group to no home anticoagulation group. Also, there was no statistical difference when each anticoagulant was compared to one another.

Shock

A total of 294 patients out of 13,440 (2.18%) patients were found to have shock associated with their upper GI bleed. For each anticoagulant studied, incidence of shock and percentage of development of shock is listed in Table 2. There was no statistical significance when comparing no anticoagulation group to patients on anticoagulants and also no significance was noted when one anticoagulant is compared to another. 

Red blood cell transfusions

The average number of packed red blood cell (pRBC) units transfused is listed in Table 3. On average, for all patients presenting with upper GI bleed, 0.8414 units of pRBC were transfused. Patients on no anticoagulation had a lower mean units of pRBC transfused (0.428) when compared to the warfarin (0.906) and DOACs group (0.958), though not statistically significant.

**Table 3:** Mean red blood cell units transfused and length of stay in patients on DOACs vs. warfarin vs. no AC. Number of patients in each group and associated standard deviations for RBC transfusions (in units) and length of stay (in days). SD: standard deviation; DOAC: direct-acting oral anticoagulation; AC: anticoagulation.

Category	Mean red blood cell units (±SD)	Mean length of stay (±SD)
DOACs	0.958 (±2.026)	4.76 (±6.759)
Apixaban	1.089 (±2.095)	5.872 (±7.547)
Dabigatran	0.8 (±1.694)	3.571 (±4.481)
Rivaroxaban	0.984 (±1.905)	4.851 (±4.477)
Warfarin	0.906 (±1.7)	6.098 ( ±7.418)
No oral anticoagulation	0.428 (±1.543)	2.566 (±5.29)

Subanalysis, seen in Table 4, showed that patients not on oral anticoagulation were found to have a statistically significant decrease in RBC unit transfusion if they did not report alcohol use, or a history of CKD, HF, AF, VTE, PVD (0.475 units, 0.549 units, 0.196 units, 0.346 units, 0.374 units, 0.303 units, respectively (p <0.05)). Linear regression analysis showed that patients not on home PPI were expected to have an RBC transfusion count 0.298 units less than patients that on home PPI (p<0.001). Furthermore, patients on DOACs and alcohol use have an average RBC transfusion count that was 0.922 units more than those without reported alcohol use (p=0.006). In the warfarin group, there was no statistical significance noted when comparing pRBC transfusions and also when comparing to baseline comorbidities. 

**Table 4:** Number of units of RBC transfused and LOS (days) when comparing exposure or lack of exposure to confounding variables seen in ours groups (no AC vs. warfarin vs. DOAC). The number of units of RBCs transfused and the LOS (in days) for each of our studied groups (no AC vs. warfarin vs. DOAC) when exposed vs not exposed to each of our confounding variables. Marked with an asterisk (*) are the statistically significant values (p<0.05). RBC: red blood cell; LOS: length of stay; AC: anticoagulation; DOAC: direct-acting oral anticoagulation; PPI: proton pump inhibitor; HTN: hypertension; CAD: coronary artery disease; COPD: chronic obstructive pulmonary disease; VTE: venous thromboembolism; DVT: deep venous thrombosis; PE: pulmonary embolus; PVD: peripheral vascular disease; CKD: chronic kidney disease.

	RBC transfusion			LOS		
Variables	No AC	Warfarin	DOAC	No AC	Warfarin	DOAC
No home PPI	-0.298*	-0.503	-0.293	-1.364*	-0.417	-0.874
Home PPI	0.000	0.000	0.000	0.000	0.000	0.000
No home aspirin	-0.057	-0.418	-0.089	-0.633*	-0.268	-0.921
Home aspirin	0.000	0.000	0.000	0.000	0.000	0.000
No home H2 blocker	0.066	0.049	0.349	-0.326	2.109	0.237
Home H2 blocker	0.000	0.000	0.000	0.000	0.000	0.000
No home P2Y12	-0.105	0.487	-0.291	-0.226	2.653	-0.830
Home P2Y12	0.000	0.000	0.000	0.000	0.000	0.000
No history of H. pylori	0.071	1.351	-5.922*	0.676	-3.509	-6.790
History of H. pylori	0.000	0.000	0.000	0.000	0.000	0.000
No home NSAIDs	-0.137*	-0.212	0.178	0.225	0.619	0.058
Home NSAIDs	0.000	0.000	0.000	0.000	0.000	0.000
No home alcohol use	-0.475*	0.141	-0.922*	-1.993*	1.202	-1.969
Home alcohol use	0.000	0.000	0.000	0.000	0.000	0.000
Female	-0.070*	-0.234	-0.222	-0.167	-1.336	-0.263
Male	0.000	0.000	0.000	0.000	0.000	0.000
No history of CKD	-0.549*	-0.134	-0.307	-2.777*	0.046	-2.730*
History of CKD	0.000	0.000	0.000	0.000	0.000	0.000
No history of CAD	-0.074	0.034	0.098	-0.624*	-2.419	0.151
History of CAD	0.000	0.000	0.000	0.000	0.000	0.000
No history of COPD	-0.046	1.269	-0.444	0.758	8.649	0.534
History of COPD	0.000	0.000	0.000	0.000	0.000	0.000
No history of heart failure	-0.196*	-0.353	-0.183	-0.526*	0.423	0.337
History of heart failure	0.000	0.000	0.000	0.000	0.000	0.000
No history of atrial fibrillation	-0.346*	-0.207	-0.236	-2.764*	-0.212	-0.529
History of atrial fibrillation	0.000	0.000	0.000	0.000	0.000	0.000
No history of VTE	-0.374*	-0.389	-0.260	-2.180*	-5.198	-1.376
History of VTE	0.000	0.000	0.000	0.000	0.000	0.000
No history of PVD	-0.303*	0.006	0.092	-1.741*	-3.473	-1.388
History of PVD	0.000	0.000	0.000	0.000	0.000	0.000
Former smoker	0.117*	0.246	-0.003	0.362*	0.913	-0.541
Current smoker	0.083*	0.443	0.045	0.451*	0.844	0.855
Unknown smoking history	-0.014	-0.174	0.601	-0.489*	0.779	5.862*
Never smoker	0.000	0.000	0.000	0.000	0.000	0.000

Length of stay

The average length of stay for patients not on oral anticoagulation was 2.56 days (Table 3). Using a linear regression model, seen in Table 4, patients not on oral anticoagulation were found to have a significantly shorter length of stay if they did not report home aspirin use, reported regular alcohol use, CKD, CAD, HF, and VTE (0.633, 1.99, 2.78, 0.62, 0.53, 2.18 days, respectively (p<0.001)). Patients on home PPI had an increased length of stay by 1.36 days (p<0.001) compared to those not on home PPI.

The average length of stay for patients on DOACs was 4.76 days and the average length of stay for the patients on warfarin was 6.098 days (Table 3). Using a linear regression model (Table 4), patients on warfarin had an increased mean length of stay when compared to DOACs (4.76 days) and no anticoagulation group (2.56 days), though not statically significant. There was one statistically significant finding noted when comparing length of stay and baseline comorbidities, such that patients without a history of CKD are expected to have a shorter length of stay (2.73 days) when compared to patients with a diagnosis of CKD (p<0.0001). During an intra-drug comparison, patients on rivaroxaban had an average length of stay 1.090 days less than patients on warfarin (p=0.0435).

## Discussion

We observed that patients on either warfarin or apixaban were less likely to die when presenting with an upper GI bleed compared to those in the no anticoagulation group. A natural assumption would be that patients on anticoagulation would have more life-threatening bleeds than those who were not on anticoagulation, but this was not the case in our study. These patients were more often co-prescribed a PPI if they were already on anticoagulation, reducing their overall risk of life-threatening gastrointestinal bleeding. As shown in our results, more than 60% of patients on either apixaban, rivaroxaban, dabigatran, or warfarin were also on a PPI. Yet, for patients on no oral anticoagulation, only 38.87% were also on a PPI. Based on these findings, we speculate that PPI medications provide a survival benefit in the setting of upper GI bleeding. 

The PPI influence remerged our subanalysis comparing DOACs, warfarin, or no anticoagulation. Our results revealed that patients not on home PPIs had higher odds of mortality in the setting of upper-GI bleeding when compared to patients with home PPI. Patients on a home PPI, irrespective of them being on anticoagulation, were less likely to die from a GI bleed, suggesting a clear advantage of home PPI use in the setting of upper-GI bleeds requiring hospital admission. It was previously reported that the use of PPI medications decreased the incidence of GI bleeds. Ray et al. found that the incidence of hospitalization for upper GI bleeding was highest in patients on rivaroxaban and lowest for those on apixaban. However, for each anticoagulant, hospitalization was lowest among patients prescribed PPI co-therapy [11]. Thus, it appears that PPI medications lead to a decreased incidence of upper GI bleeds and also provide a mortality benefit if they do occur.

Incidence of shock and acute renal failure were also examined in our study and we found no significant differences between patients on DOACs versus patients not on an anticoagulant. Our analyses of RBC transfusions revealed that patients not on an anticoagulant had lower RBC transfusion requirements when compared to the warfarin and DOACs group. In addition, patients not on an anticoagulant and not on home PPI had a significantly higher RBC transfusion requirement than those on home PPI. Patients who used alcohol, have a history of AF, HF, and had a history of CKD required on average more units of RBCs than those who did not have these conditions. These findings are in agreement with Mellemkjaer et al., Kärkkäinen et al., and Liang et al. It has been well studied that each of these risk factors poses a higher risk for UGIB and as seen in our study, require more units of RBCs [12-14].

Limitations

Our study had several limitations. Patients were identified using discharged ICD-10 codes through an electronic administrative database using the HCA databank. The accuracy of the ICD-10 codes is dependent on multiple factors including but not limited to communication quality between physicians and patients, clinicians’ expertise and precision of diagnoses and coders’ experience and attention to choosing the best code. Hence, as with any administrative database, some inaccuracies and variations in recording and documentation exist. Also, there were a small number of patients on dabigatran in our study, leading to potential underpowered results. 

## Conclusions

In our retrospective cohort study, we studied a control group of patients not on anticoagulation and compared it to patients on DOACs and warfarin. Overall, there were no statistically significant findings noted when looking at the development of shock and acute renal failure in this patient population. We found that patients on home PPI were less likely to die if admitted with upper GI bleeds regardless of the use of oral anticoagulation. We also found that patients with reported alcohol use and a history of CKD required more RBC transfusions and had a longer length of stays. Our findings support these comorbidities being risk factors for worse outcomes. These results can help clinicians (both in the emergency department and on medical floors) when risk stratifying patients for further management such as endoscopy. Future studies should focus on specific interventions to try to decrease mortality in patients without home use of PPI such as the timing of endoscopy or admissions to higher levels of care. 
